# Reconstruction of Stage IV Pressure Ulcers Using Delayed Flaps in a Pediatric Patient With Spinal Cord Injury: A Case Report

**DOI:** 10.7759/cureus.102840

**Published:** 2026-02-02

**Authors:** Audrey Ulfers, Genevieve Messa, Kory Imbrescia, Mohamad Masoumy

**Affiliations:** 1 Department of Plastic and Reconstructive Surgery, Louisiana State University Health Sciences Center, New Orleans, USA; 2 Department of Plastic and Reconstructive Surgery, Tulane University School of Medicine, New Orleans, USA

**Keywords:** delayed flap, multidisciplinary decision-making, pediatrics, pressure ulcers, spinal cord injury

## Abstract

Reconstructing pressure ulcers (PUs) in pediatric patients with spinal cord injury (SCI) requires careful surgical planning to minimize functional loss. This case report presents a seven-year-old female patient with a complete T3 SCI and paraplegia who developed large stage IV PUs over the bilateral anterolateral hips. Reconstruction of the right hip was successfully performed using a delayed fasciocutaneous flap based on the superficial inferior epigastric artery/vein (SIEA/SIEV), which was selected as an alternative to a traditional musculocutaneous flap to minimize core muscle function loss for the patient. The positive outcome of the present case demonstrates the potential of delayed flaps for reconstructing large wounds while preserving muscle function in pediatric patients with SCI.

## Introduction

Pressure ulcers (PUs), also known as decubitus ulcers, are localized injuries to the skin and underlying tissues caused by prolonged pressure, which restricts blood flow to the affected area. The resulting compromised perfusion leads to ischemia, tissue damage, and necrosis [1]. These wounds present a significant challenge for individuals with paralysis, particularly those with spinal cord injuries (SCIs) who are often unable to reposition themselves to relieve pressure [1, 2]. Over time, sustained pressure results in the destruction of soft tissue, leading to deep, often chronic wounds that are difficult to treat [1]. PUs not only compromise tissue integrity but also significantly affect the patient’s quality of life. Individuals with SCI and PUs report lower levels of well-being, greater health problems, and limitations in physical and social activities [2, 3]. 

PUs are highly prevalent among SCI patients, with a global pooled prevalence of 32.36% and a lifetime prevalence reaching up to 85.7% [4, 5]. Additionally, PUs are associated with increased morbidity and mortality rates [6]. Complications include infections, sepsis, necrotizing fasciitis, gangrene, osteomyelitis, myonecrosis, amyloidosis, and hypoproteinemia with worsened malnutrition [1].  Furthermore, SCI patients with chronic PUs experience an estimated 50.3% reduction in life expectancy, highlighting the severe systemic impact of these wounds and the need for timely and effective management [7]. Healthcare costs associated with treating PUs are substantial, imposing an annual economic burden of up to $17.8 billion in the United States alone [8]. In SCI patients with stage III-IV PUs, a Canadian study estimated a cost of $175,198 per hospitalization [9]. 

A 2014 study found that PUs significantly impact the quality of life in individuals with SCI. The study showed that 65.3% of participants reported reduced activity levels, and those with PUs experienced greater dissatisfaction with daily activities compared to those without. Self-reported measures highlighted the physical, psychological, and social limitations caused by PUs, demonstrating their severe effect on well-being [2]. 

While various treatment strategies exist for managing PUs, including local wound care and advanced dressings, surgical interventions like musculocutaneous flaps are often considered the gold standard for complex cases where bone or critical structures are exposed  [10]. In recent years, the concept of delayed flaps has reemerged as a promising alternative to traditional muscle flaps. The approach incorporates a staged technique in which part of the flap’s blood supply is intentionally disrupted during an initial operation to allow the tissue time to develop a more robust vascular network before final inset [11]. This mechanism, known as the delay phenomenon, has been well-documented in other reconstructive surgeries, including breast reconstruction, where it has been shown to improve flap survival [11]. 

The advantages of delayed flaps may be particularly critical in SCI patients, who often have compromised circulation that impairs wound healing. The American Spinal Injury Association states that unrelieved pressure causes poor perfusion in SCI patients, promoting wound chronicity [12].  SCI often results in venous vascular dysfunction distal to the level of injury, potentially impairing tissue fluid homeostasis, lymphatic contractility, and connective tissue integrity; these factors are critical to wound healing, and their dysfunction may contribute to wound healing disorders [13]. 

One of the key advantages of delayed flaps is the ability to use larger, better-vascularized tissue for reconstruction. In PU reconstruction, increased flap vascularity allows for better tissue coverage, reduces the risk of flap failure, and ensures more reliable functional restoration. Furthermore, delayed flaps offer the flexibility of more tailored reconstruction, as the size and shape can be precisely matched to the patient's specific needs [14]. 

This case report discusses the successful application of the delayed flap technique in a patient with stage IV PUs resulting from a complete SCI at the T3 vertebral level, demonstrating its potential as an effective reconstructive strategy in complex and challenging cases. 

## Case presentation

**Figure 1:**
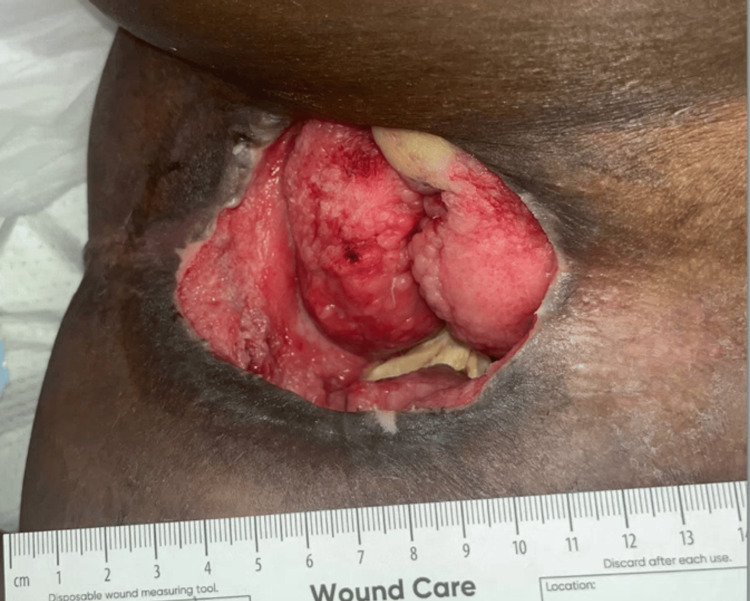
Initial presentation of the right anterolateral hip pressure ulcer at our institution

A seven-year-old female suffered gunshot wounds to the left anterior neck and right anterior chin, resulting in complete paralysis below the T3 vertebral level and rendering her wheelchair dependent. Three years after the initial injury, the patient presented to our institution following transfer from an outside hospital for progressively worsening PUs and osteomyelitis. On presentation, she had large stage IV PUs in the bilateral anterolateral hip region over the anterior superior iliac spine (ASIS) and femur. She required a Broviac catheter for intravenous antibiotics targeting multidrug-resistant organisms, and her albumin was low at 2.3 g/dL, indicating compromised nutritional status. According to her mother, the ulcers were caused by excessively tight compression stockings and had likely been present for several weeks prior to initial evaluation at an outside hospital (Figure 1). 

**Figure 2:**
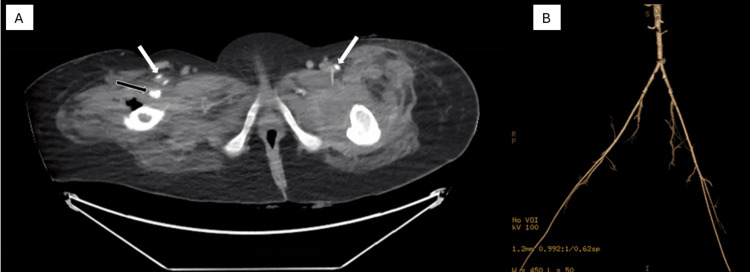
Preoperative computed tomography angiography (CTA) images showing patent major arteries without arterial abnormality in the inguinal regions A) Axial CTA showing the bilateral superficial femoral arteries (white arrows) and the right profunda femoris artery (black arrow). B) Angiographic three-dimensional volume-rendered image showing arterial patency.

Initial treatments included advanced wound care, aggressive debridement, bilateral girdle stone procedure, and repositioning techniques, all of which were unsuccessful. Despite receiving treatment for approximately one year at an outside hospital, her PUs showed minimal improvement. Given the chronicity and severity of the patient's condition, a multidisciplinary evaluation at our institution selected a delayed fasciocutaneous flap technique due to concerns of compromising essential muscle function in a paraplegic patient and prior exhaustion of other options. Prior to reconstruction, she required minimal to moderate assistance with wheelchair transfers and basic mobility. Computed tomography angiography demonstrated patent common femoral, superficial femoral, and profunda femoris arteries without abnormality (Figure 2).

**Figure 3:**
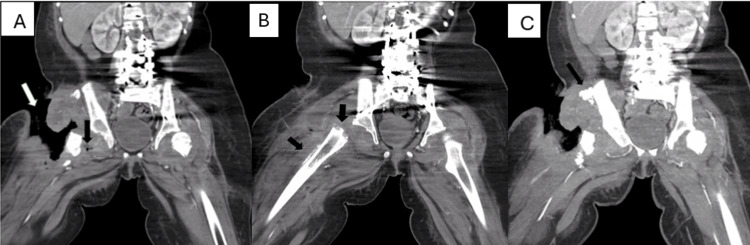
Preoperative coronal computed tomography (CT) images of the bilateral lower extremities, highlighting the extent of the ulcer and anatomical concerns affecting management A) Right inguinal ulcer extending to the proximal femur (white arrow); soft tissue loss and deep soft tissue air medial to the proximal right femur (black arrow). B) Mild periosteal reaction of the right femur (black arrows). C) Heterotopic ossification adjacent to the right iliac crest (black arrow).

However, extensive soft tissue loss, deep soft tissue air (Figure 3A), periosteal reaction suggesting osteomyelitis in the right proximal femur (Figure 3B), and heterotopic bone formation adjacent to the right iliac crest (Figure 3C) rendered the anterior thigh unsuitable for flap reconstruction. Additionally, the anterior compartment lower extremity muscles originating from the ASIS were necrotic, leaving a large cavity in this region and further limiting reconstructive options (Figure 3C).

The right hip wound measured 13.0 cm x 9.0 cm x 5.5 cm with exposed bone.  A pedicled superficial inferior epigastric artery/vein (SIEA/SIEV) flap was elevated and delayed in a staged fashion to enhance perfusion prior to final inset. The flap measured approximately 20.0 cm in length from the pedicle, 15.0 cm laterally from the fulcrum, and 8.0 cm at its widest point.  A seven-day vascular delay was chosen, as this duration falls within the established optimal time frame, approximately seven to 14 days, recommended in the literature [15]. Superior and inferior incisions were made, and the distal skin bridge (~5.0 cm wide) was initially preserved while the flap was undermined medially, leaving the pedicle intact. In a subsequent stage, four days later, the distal bridge was partially divided to further stress the flap and promote vascular recruitment.  Three days later, the flap was mobilized. Prior to the final inset, the flap was temporarily positioned in a “mimicked inset,” and the absence of distal congestion confirmed sufficient perfusion. Doppler assessment confirmed strong arterial and venous signals in both the SIEA and SIEV proximally, and both bright red arterial bleeding and venous drainage were observed at the distal edges, indicating adequate inflow and outflow. Due to the thickness and bulk of the flap and misalignment of the skin edges medially and laterally, a portion of the distal flap was de-epithelialized and buried into the wound bed to obliterate dead space. This approach allowed for improved contour and reduction of potential fluid accumulation.  In contrast, initial management of the left hip defect with negative pressure wound therapy resulted in adequate granulation tissue and delineation of a more limited soft tissue defect. Based on the smaller defect size and reduced dead space compared to the right hip, reconstruction was performed using a rectus femoris muscle flap, followed by split-thickness skin grafting.

**Figure 4:**
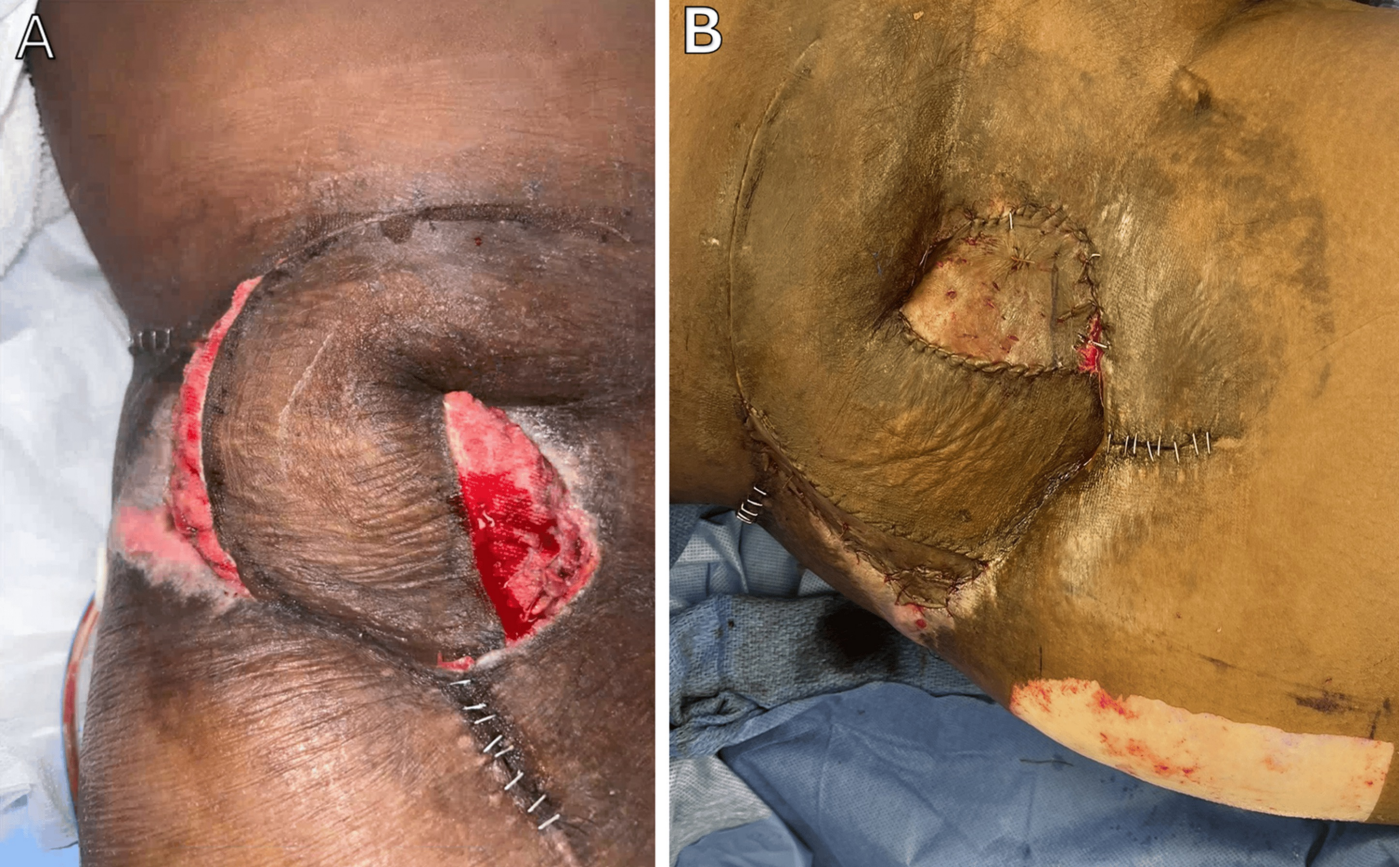
Postoperative presentation of the right anterolateral hip pressure injury after superficial inferior epigastric artery/vein (SIEA/SIEV) flap reconstruction with a seven-day delay (A) Delayed right SIEA/SIEV flap immediately following surgery. (B) Delayed right SIEA/SIEV flap at one week postoperatively.

Postoperatively, the delayed SIEA/SIEV flap healed well and developed healthy granulation over the exposed adipose tissue (Figure 4A). The flap maintained excellent vascular perfusion, confirmed via Doppler signals. Within three weeks, the entire skin graft on the medial and lateral surfaces of the delayed flap had integrated well (Figure 4B).

The donor site also healed appropriately but developed increased serous drainage after Jackson-Pratt drain removal, requiring frequent dressing changes. Hypergranulation tissue developed at the raw surfaces of the flap-native skin interface and was treated with silver nitrate to promote epithelialization. Approximately one month after the final inset, the patient developed persistent drainage from the right groin, concerning for a synovial leak with the formation of a fistulous tract. She underwent interventional radiology-guided drain placements and betadine sclerotherapy with doxycycline for three weeks, after which output declined, and she remained clinically stable. She was discharged after a total inpatient stay of 176 days. At discharge, she demonstrated independent static sitting balance, minimal to moderate assistance with wheelchair transfers, and wheelchair propulsion up to 50 feet, without new truncal or upper extremity functional deficits. Since discharge about two years ago, she has had no complications and no recurrence of PUs or flap-related complications at the SIEA/SIEV flap site. 

## Discussion

Managing PUs in pediatric patients with SCIs requires innovative and tailored approaches due to the complex nature of these wounds. This case underscores the advantages of the delayed flap technique, particularly in patients with paraplegia and SCI, in whom preserving muscle function is critical to quality of life. 

Traditional musculocutaneous flaps remain the gold standard for reconstructing large, complex PUs [10]. However, their use in SCI patients presents unique challenges, particularly in pediatric cases where long-term mobility and essential muscle function are primary considerations. In this patient, a rectus abdominis muscle flap was initially considered but ultimately rejected to preserve maximal core stability. Given the patient’s paraplegia, maintaining core strength was essential for future mobility, posture, and respiratory function. The decision aligns with recommendations from Sgarzani et al., who emphasize that flap selection in SCI patients should prioritize not only muscle preservation but also long-term reconstructive planning, including sparing donor sites for potential future recurrence [14]. Recent evidence from a 2021 systematic review of PU reconstruction highlights that while musculocutaneous flaps tend to have the lowest recurrence rates at 6.3% but a complication rate of 18.2%, fasciocutaneous and perforator flaps may be suitable as first-line options in select cases, particularly when preserving muscle function is critical [16]. Notably, the literature also shows that delayed fasciocutaneous flaps are understudied in the context of PU management, indicating a gap in understanding their long-term outcomes and recurrence rates. 

The patient’s surgical history and anatomical constraints further complicated flap selection. Extensive necrosis at the origins of the lower extremity muscles from the ASIS, in combination with vascular compromise from prior surgeries, made anterior thigh-based flaps an unfavorable option. Additionally, the location of the PUs and the risk of compromised perfusion with hip flexion rendered free flaps impractical. Hip flexion could impinge on the vascular pedicle, causing anastomotic compromise and increasing the risk of flap failure. These constraints reinforced the decision to use a delayed SIEA/SIEV flap, which provided a well-vascularized, reliable tissue source without sacrificing critical muscle function. 

The success of the delayed flap approach in this case highlights the benefits of the delay phenomenon, a well-established principle in reconstructive surgery. By temporarily interrupting vascular inflow while maintaining venous drainage, the delay technique induces neovascularization of the flap, likely by stimulating both angiogenesis and vasculogenesis [14, 17]. Furthermore, the delay phenomenon has been found to alter the function of neutrophils, providing anti-inflammatory benefits [18]. This physiologic benefit has been demonstrated across various flap types. For example, a recent study found that surgical delay significantly increased survival of expanded random-pattern flaps in children by 73%, supporting the general utility of delay in enhancing flap viability [19]. In this patient, delaying the SIEA/SIEV flap for seven days allowed for enhanced vascularization, confirmed intraoperatively using Doppler assessment. This strategy mitigated risks associated with poor circulation, a common concern in SCI patients who often have impaired microvascular function [13, 20]. 

Despite the advantages of delayed flaps, complex reconstructions remain vulnerable to postoperative challenges. In this case, wound healing complications on the left side warranted more extensive care. This highlights the importance of continuous monitoring and proactive wound management in pediatric SCI patients. Early identification of potential complications, such as infection or impaired wound healing, can significantly improve long-term outcomes [20]. 

## Conclusions

This case illustrates how a delayed flap can be used as a reconstructive option in a pediatric SCI patient with large, refractory PUs. In this patient, the staged approach supported flap perfusion and enabled closure while preserving key muscle groups vital to the patient’s long-term mobility and core stability. However, conclusions about the broader reliability or comparable effectiveness of delayed flaps are limited by the single-patient design. Large studies with longer-term outcomes are needed to better define the role of delayed flaps in pediatric PU reconstruction and their impact on recurrence and function.
